# First Diagnostic Marine Reptile Remains from the Aalenian (Middle Jurassic): A New Ichthyosaur from Southwestern Germany

**DOI:** 10.1371/journal.pone.0041692

**Published:** 2012-08-01

**Authors:** Erin E. Maxwell, Marta S. Fernández, Rainer R. Schoch

**Affiliations:** 1 Staatliches Museum für Naturkunde, Stuttgart, Germany; 2 Museum für Naturkunde, Leibniz-Institut für Evolutions- und Biodiversitätsforschung, Berlin, Germany; 3 Departamento Paleontología de Vertebrados, Museo de La Plata, La Plata, Argentina; Raymond M. Alf Museum of Paleontology, United States of America

## Abstract

**Background:**

The Middle Jurassic was a critical time in the evolutionary history of ichthyosaurs. During this time interval, the diverse, well-studied faunas of the Lower Jurassic were entirely replaced by ophthalmosaurids, a new group that arose sometime prior to the Aalenian-Bajocian boundary and by the latest middle Jurassic comprised the only surviving group of ichthyosaurs. Thus, the Middle Jurassic Aalenian-Bathonian interval (176–165 million years ago) comprises the time frame during which ophthalmosaurids not only originated but also achieved taxonomic dominance. However, diagnostic ichthyosaur remains have been described previously from only a single locality from this interval, from the Bajocian of Argentina.

**Methodology/Principal Findings:**

In this paper, we describe a new species of ichthyosaur based on a partial articulated specimen from the Middle Jurassic of southwestern Germany. This specimen was recovered from the Opalinuston Formation (early Aalenian) and is referable to *Stenopterygius aaleniensis* sp. nov. reflecting features of the skull and forefin. The genus *Stenopterygius* is diverse and abundant in the Lower Jurassic of Europe, but its presence has not previously been confirmed in younger (Middle Jurassic) rocks from the northern hemisphere.

**Conclusions/Significance:**

This specimen represents the only diagnostic ichthyosaur remains reported from the Aalenian. It bears numerous similarities in size and in morphology to the Lower Jurassic species of the genus *Stenopterygius* and provides additional evidence that the major ecological changes hypothesized to have occurred at the end of the Toarcian took place sometime after this point and most likely did not occur suddenly. There is currently no evidence for the presence of ophthalmosaurids in the northern hemisphere during the Aalenian-Bathonian interval.

## Introduction

The Middle Jurassic was a critical time in the evolutionary history of ichthyosaurs: during this time interval, the diverse, well-studied faunas of the Lower Jurassic were entirely replaced by ophthalmosaurids, a new group that arose sometime prior to the Aalenian-Bajocian boundary [1], and by the latest middle Jurassic (Callovian) comprised the only surviving ichthyosaur clade [2]. Thus, the Middle Jurassic Aalenian-Bathonian interval (176–165 million years ago) comprises the time frame during which ophthalmosaurids not only originated but also achieved taxonomic dominance. However, the Middle Jurassic is extremely poorly documented from the standpoint of marine reptile paleontology [3], [4]. Excluding the Callovian-aged Oxford Clay Formation, which has produced a diverse marine reptile fauna including ichthyosaurs [5], [6], diagnostic ichthyosaur remains have been described from only a single Middle Jurassic locality – the Bajocian of Chacaico Sur, Argentina. This site has produced two ichthyosaur taxa, each known from a single incomplete specimen [7], [8]: the basal ophthalmosaurine (sensu [9]) *Mollesaurus periallus* Fernández, 1999, and *Chacaicosaurus cayi* Fernández, 1994. *Chacaicosaurus cayi* is considered to be referable to the Lower Jurassic genus *Stenopterygius* (see [10]), but disagreement exists on this point: some authors consider *S. cayi* to be intermediate between *Stenopterygius* and Ophthalmosauridae [9], [11].

Fragmentary ichthyosaurian material is not abundant in the Aalenian-Bathonian interval, but isolated rostral fragments, teeth, and vertebrae have been reported from the early Aalenian to late Bajocian of Germany [12]–[14], earliest Aalenian and Bathonian of France [14], [15], early Bajocian of western Argentina [16], early Bathonian of Russia [17], and Bathonian of the UK [18]. McGowan [19] concluded that none of these Middle Jurassic finds were diagnostic. However, anecdotal reports indicate more complete ichthyosaur material from the lower Aalenian of southwestern Germany [8], [11]. The affinities of these specimens are of great interest, because no diagnostic ichthyosaur material is described from the Aalenian [2], and no Lazarus species span this gap.

## Materials and Methods

**Figure 1:**
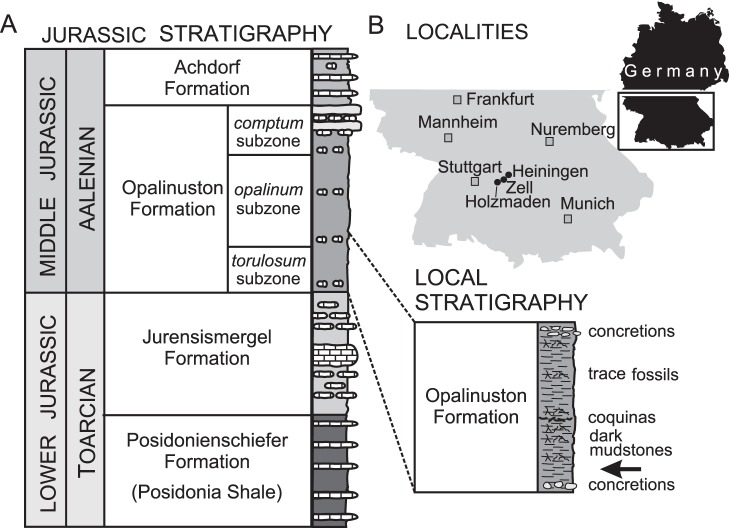
Geographic and stratigraphic information. A, Stratigraphic section modified from Geyer et al. [22]; the location of the specimen is indicated in the inset by an arrow. B, location of the Zell and Heiningen localities, Baden-Württemberg, Germany; Holzmaden, the classic early Toarcian *Stenopterygius* locality, is also indicated.

**Figure 2:**
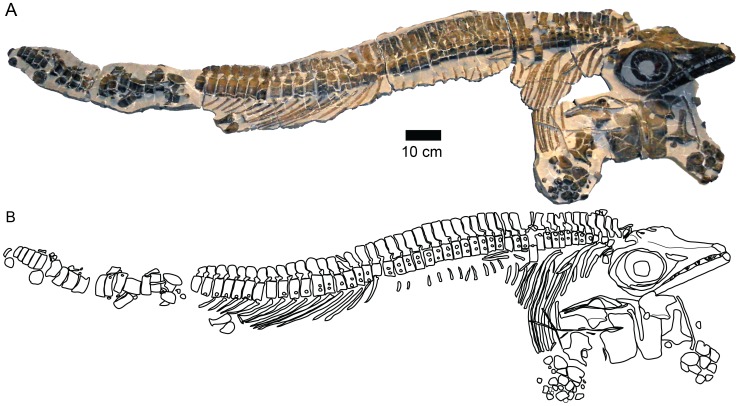
SMNS 90699, holotype, *Stenopterygius aaleniensis* sp. nov.

In 1976, an articulated ichthyosaur was discovered in a hard limestone concretion of the basalmost Opalinuston Formation, 700 m SE of Zell near Göppingen (Baden-Württemberg) (Fig. 1). It was prepared by Michael Maus in 2005–07. The specimen (SMNS 90699), an ichthyosaur prepared in right lateral view, is preserved in three dimensions (Fig. 2). Some general measurements are presented in Table 1. The skull is oriented at approximately a 90° angle to the rest of the body, and the rostrum is broken anterior to the external nares. Both the right and left sides of the skull have been prepared. The vertebral column and ribs are also three-dimensionally preserved. The cervical region is disrupted, and slight disruption also occurs in the anterior thoracic region. Disarticulation is significant in the anterior caudal region, and the column is truncated prior to the apical region. Few ribs are preserved in their entirety, and a large gap is present in the mid torso. Only a few gastralia are present; these have been displaced and are located around the pectoral girdle. The torso, measured from the posterior skull to the pelvic girdle, is approximately 102 cm in length. The coracoids, interclavicles, right clavicle and scapulae are exposed in ventral view. Only the proximal-most elements are in articulation, and most of the distal paddle is missing. The left paddle is visible in ventral view, and the right in dorsal view. The hind limb and pelvic girdle are only represented by the proximal-most femur and the end of a second element. The specimen is currently on display in the public gallery.

**Table 1:** General measurements (SMNS 90699).

	Length (mm)
Lower jaw	280 (broken anteriorly)
Sclerotic ring – internal diameter	39/41 (height/length)
Sclerotic ring – external diameter	95 (length)
Orbital length	118/103 (right/left)
Orbital height	101/107 (right/left)
Narial length	51 (right)
Dorsal length (posterior skull–pelvis)	1020

A second, less well-preserved specimen (SMG uncatalogued) was discovered in the early 1970s in the commercial brick quarry Mohring at Heiningen, again in the basalmost Opalinuston [20] (Fig. 1). This is on display at the Natural History exhibition of the Städtisches Museum Göppingen. It consists of a partial skull (the same portion as in SMNS 90699), and preflexural vertebral column (Fig. S1). The neural spines are preserved in full over a short stretch of the mid-dorsal region, otherwise they have been eroded dorsally. No ribs are preserved in their entirety, but the proximal segments are present through most of the torso. Based on vertebral measurement data (Table S1, Fig. S2), SMG uncatalogued is smaller than SMNS 90699, though within the size range of adult *S. quadriscissus.*

### Geological Setting

The two localities that produced the specimens described here are only 5 km apart, both falling within the basalmost Opalinuston Formation (Fig. 1). This forms the basal unit of the Middle Jurassic in southern Germany (Dogger α, *opalinum* Zone, *torulosum* Subzone) [21]. When actively quarried, the Heiningen Brick Quarry (Ziegelwerk) exposed the lower 10 m of the Opalinuston Formation. This monotonous series of dark, poorly laminated claystones was deposited in a oxic epicontinental marine basin [22]. Sedimentation rates and subsidence were relatively high, and the fauna includes soft-bottom dwellers (bivalve *Bositra buchi*, gastropods *Teretrina opalina* and “pelikan foot” *Toarctocera subpunctata*), ammonites (*Leioceras opalinum*, *Pachylytoceras torulosum*), brachiopods (*Discina* sp.), and plankton (coccoliths, radiolarians, and dinoflagellates) [20], [22]. Driftwood is common. Vertebrate remains such as ganoid fishes (*Dapedium* sp.) and ichthyosaur vertebrae are rare, but in southern Germany the general lack of Aalenian reptiles is caused by the rare exposure of Middle Jurassic rocks rather than preservational bias. However, the fast sedimentation rates of the Opalinuston Formation (120–150 m for only the Lower Aalenian) suggest that vertebrate finds should be fewer per cubic meter than, for instance, in the Toarcian Posidonia Shale. At Zell and Heiningen, the articulated ichthyosaurs were found in hard, sideritic limestone concretions.

### Institutional Abbreviations

PMU, Evolutionsmuseet Paleontologi, Uppsala Universitet; MHH, Museum Hauff, Holzmaden; MOZ, Museo Olsacher, Zapala, Argentina; SMG, Städtisches Museum Göppingen, Germany; SMNS, Staatliches Museum für Naturkunde Stuttgart, Germany.

### Nomenclatural Acts

The electronic version of this document does not represent a published work according to the International Code of Zoological Nomenclature (ICZN), and hence the nomenclatural acts contained in the electronic version are not available under that Code from the electronic edition. Therefore, a separate edition of this document was produced by a method that assures numerous identical and durable copies, and those copies were simultaneously obtainable (from the publication date noted on the first page of this article) for the purpose of providing a public and permanent scientific record, in accordance with Article 8.1 of the Code. The separate print-only edition is available on request from PLoS by sending a request to PLoS ONE, Public Library of Science, 1160 Battery Street, Suite 100, San Francisco, CA 94111, USA along with a check for $10 (to cover printing and postage) payable to “Public Library of Science”.

In addition, this published work and the nomenclatural acts it contains have been registered in ZooBank, the proposed online registration system for the ICZN. The ZooBank LSIDs (Life Science Identifiers) can be resolved and the associated information viewed through any standard web browser by appending the LSID to the prefix “http://zoobank.org/”. The LSID for this publication is: urn:lsid:zoobank.org:pub:61C49889-7098-4EFA-B7E9-16533DDAB143.

## Results

### Systematic Paleontology

Ichthyosauria de Blainville, 1835.

Parvipelvia Motani, 1999.

Genus *Stenopterygius* Jaekel, 1904.

#### Type species

Stenopterygius quadriscissus (Quenstedt, 1856).

Stenopterygius aaleniensis sp. nov.

urn:lsid:zoobank.org:act:DAEF2283-FDB2-4357-A2BE-0A7739DC3267.

**Figure 3:**
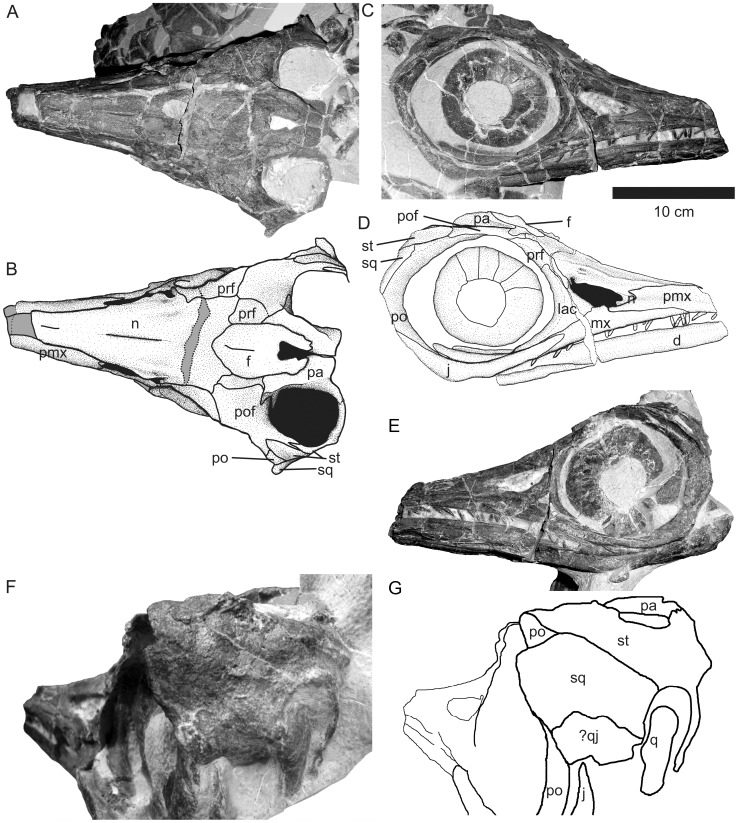
Skull of SMNS 90699. A, B, Dorsal view; C, D, Right lateral view; E, Left lateral view; F, G, Temporal region in posterolateral view. Abbreviations: d, dentary; f, frontals; j, jugal; lac, lacrimal; mx, maxilla; n, nasals; pa, parietal; pmx, premaxilla; po, postorbital; pof, postfrontal; prf, prefrontal; q, quadrate; qj, quadratojugal; sq, squamosal; st, supratemporal.

**Figure 4:**
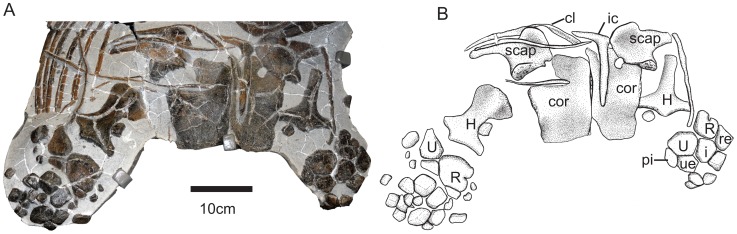
Pectoral girdle of SMNS 90699. Abbreviations: cl, clavicle; cor, coracoid; H, humerus; i, intermedium; ic, interclavicle; pi, pisiform; R, radius; re, radiale; scap, scapula; U, ulna; ue, ulnare.

**Figures** 2, 3, and 4.

#### Holotype

SMNS 90699 (Fig. 2), an articulated specimen of a mature adult preserving the posterior skull, presacral vertebral column, pectoral girdle and proximal forelimbs.

#### Locality and horizon

Road-cut 700 m SE of the center of Zell am Aichelberg, along the road to Bad Boll (Kreis Göppingen, Baden-Württemberg, Germany). The locality was open only for a short time in June 1976 and exposed weathered Opalinuston mudstones, as confirmed by ammonites.

#### Distribution

Lower Aalenian (*opalinum* zone, *torulosum* subzone).

#### Etymology

The specific epithet reflects the stratigraphic provenance of the holotype.

#### Diagnosis

The species is referred to *Stenopterygius* to the exclusion of other Toarcian ichthyosaurs based on the following combination of shared characters: smaller anterolateral exposure of the parietals and larger anteromedial expansion of the postfrontals than in *Hauffiopteryx*; larger, more ovate upper temporal fenestrae than *Hauffiopteryx* and *Eurhinosaurus*, such that the parietal foramen is situated medial rather than anterior to the upper temporal fenestrae; parietal foramen surrounded almost entirely by the frontals (differs from *Suevoleviathan*, *Temnodontosaurus*, shared with *Hauffiopteryx*); temporal region reduced and oriented posterodorsally (differs from *Temnodontosaurus*, *Suevoleviathan*); lower jaw not strongly reduced (differs from *Eurhinosaurus*); small teeth with unornamented enamel (shared with *Eurhinosaurus* and *Hauffiopteryx*); large rectangular anteroposteriorly elongate coracoids (absent in *Suevoleviathan, Temnodontosaurus*, and *Eurhinosaurus*); dorsal process of humerus not plate-like and absence of a digit anterior to the radius (shared with all non-ophthalmosaurid ichthyosaur genera); notching of anterior edge of radius (absent in *Suevoleviathan* and Ophthalmosauridae, but shared with *Temnodontosaurus*, *Hauffiopteryx*, and some specimens of *Eurhinosaurus*); ulnare with two strongly developed equal sized distal articular facets (shared only with *Hauffiopteryx*).

Relative to all other species of *Stenopterygius:* reduced internasal depression, medial concavity between the frontals absent (present in other *Stenopterygius* species), upper temporal fenestrae ovate to rounded (generally anteroposteriorly longer and narrower in other *Stenopterygius* species), temporal region posterolaterally directed (as in *Stenopterygius quadriscissus* and *S. triscissus*), maxilla extends as far posteriorly as lacrimal (shared with all species of *Stenopterygius*), suborbital tooth positions present in adult (absent in *S. quadriscissus, S. triscissus* from the Posidonia Shale, and *S. uniter*), posterior edge of the external narial opening dorsally deflected (dorsal deflection minimal to absent in *Stenopterygius quadriscissus, S. triscissus,* and *S. uniter*), contact between radius and ulna short (longer in *S. quadriscissus*, *S. triscissus,* and *S. uniter*), anterior edge of radiale unnotched (notched in all other *Stenopterygius* species), phalanges as dorsoventrally thick as proximodistally long (as in *Stenopterygius quadriscissus* and *S. cayi*).

### Description

#### External nares

The external nares are framed by the premaxilla, maxilla, lacrimal and nasal (Fig. 3C–E). They have an elongate shape, and the posterior half is slightly deflected dorsally relative to the anterior half. A small embayment in the posterior dorsal corner is formed by the nasal.

#### Orbit

The orbit is large and rounded. It is surrounded by the lacrimal, jugal, postorbital, postfrontal, and prefrontal (Fig. 3C–E). A well-developed circumorbital area surrounds much of the orbit, with the exception of the prefrontal and postfrontal contributions.

#### Parietal foramen

In dorsal view, the parietal foramen is framed mostly by the frontals, with a small parietal contribution posteriorly (Fig. 3A–B). It is roughly triangular in shape, with a flattened anterior edge and an attenuated posterior margin. Its anterior edge is situated between the anterior margins of the upper temporal fenestrae.

#### Upper temporal fenestrae

The upper temporal fenestrae are surrounded by the postfrontal, supratemporal and parietal. They are rounded to oval in shape, and relatively large in size (Fig. 3A–B).

#### Premaxilla

The rostrum is broken anterior to the external nares. As preserved, the premaxilla is not exposed on the dorsal surface of the skull (Fig. 3A–B) but forms the lateral sides of the snout. Dorsally, it overlies the ventrolateral nasal, and posteriorly and ventrally it overlies the maxilla. The premaxilla extends posteriorly to the anterior margin of the external narial opening. The subnarial process is relatively well developed, although it does not contact the anterior process of the lacrimal (Fig. 3C–E). In contrast, the supranarial process is reduced and does not form the dorsal margin of the external nares. A few premaxillary teeth are preserved near the broken anterior end of the rostrum.

#### Maxilla

The maxilla is dentigerous and shows relatively extensive lateral exposure, extending anterior to the external narial opening and posteriorly as far under the orbit as the suborbital process of the lacrimal (Fig. 3C–E). In lateral view, it is roughly triangular in shape, and its widest point is at the midpoint of the external narial opening. The maxilla makes a small contribution to the ventral edge of the external nares. The maxillary component of the rostrum is mediolaterally wider than the mandibular component, and so the posterior maxillary teeth overlap the dentary.

#### Nasal

The internasal suture has been obliterated over much of the rostrum, and the internasal foramen is completely absent. The internasal depression is extremely reduced (Fig. 3A–B). The nasal forms the dorsal edge of the external narial opening. In the area of the posterodorsal external nares, the nasal forms a thin shelf overhanging the external narial opening. This shelf is posteriorly truncated by a notch in the ventral edge of the nasal, which is capped by a thickened ridge. Posterior to this notch, the nasal excludes the prefrontal from the external narial opening via a narrow descending process. Dorsal to the notch is a depression in which are located two small pits, and medial to these the nasal forms a supranarial ridge (Fig. 3C–E). On the dorsal skull roof, the nasals contact the prefrontals laterally. The posterior nasals contact the frontals, the prefrontals, and also have a small postfrontal contact at the posterolateral corners (Fig. 3A–B).

#### Lacrimal

The lacrimal is weakly triradiate, with the maxillary process being the least well-developed. The lacrimal forms the posteroventral and posterior margins of the external narial opening, and the anterior and anteroventral margins of the orbit. A sharp ridge separating the orbital contribution from the anterolateral contribution defines the circumorbital area. The nasal process of the lacrimal contacts the nasal anterodorsally and the prefrontal posterodorsally. The orbital process lies dorsal to the jugal, and the anterior process and the main body of the lacrimal are situated dorsal to the maxilla (Fig. 3C–E).

#### Prefrontal

The prefrontal forms the anterodorsal edge of the orbit and is excluded from the external narial opening by a thin descending process of the nasal (Fig. 3C–E). Posteriorly, the prefrontal contacts the postfrontal and also has a second medial exposure on the dorsal skull roof, which separates the postfrontal from contact with the frontals (Fig. 3A–B).

#### Frontals

The frontals are fused. They have a raised, convex profile and are flanked laterally by a flat area located anterior to the temporal fenestra. The frontals make up almost the entire margin of the parietal foramen in dorsal view, with the parietals forming only the posteriormost edge (Fig. 3A–B). The posteromedial frontal processes flanking the parietal foramen are extremely robust and appear to overlie the parietal. The contribution of the frontals to the dorsal skull roof lateral to the parietal foramen is much greater than that seen in *Stenopterygius triscissus* [23], [24].

#### Postfrontal

The postfrontal is a large element forming the anterior and lateral margins of the supratemporal fenestra. In dorsal view, it is broadly expanded anteriorly and narrows posteriorly where it forms a complex suture with the supratemporal (Fig. 3A–B). The postfrontal contacts the postorbital on its ventrolateral surface.

#### Parietal

The parietal makes up the medial edges of the upper temporal fenestra and also provides a small contribution to the anterior ventral surface via a small flange. Anteriorly, the parietal provides a small contribution to the lateral walls of the parietal foramen. Lateral to the parietal foramen, the parietal forms a shelf of bone that overhangs the anterior and medial temporal fenestra. Posteriorly, this shelf regresses medially and its posterior end is obscured by matrix. The short supratemporal process of the parietal is preserved on the left side (Fig. 3A–B).

#### Supratemporal

The supratemporal is triradiate. The lateral ramus forms an interdigitating suture with the postfrontal anteriorly and overlaps the postorbital ventrally. The supratemporal forms a small portion of the posterolateral margin of the supratemporal fenestra but is excluded anteriorly by a process of the postfrontal (Fig. 3A–B). The ventral margin of the lateral process of the supratemporal contacts the squamosal (Fig. 3C–F). The parietal ramus extends anteromedially. The ventral ramus, only partially exposed, forms the medial wall of the deeply inset facet for articulation with the quadrate (Fig. 3F–G).

#### Squamosal

Only the dorsal half of the lateral temporal region is visible in lateral view (Fig. 3C–E). The squamosal is roughly triangular in shape in lateral view. It is an anteroposteriorly and dorsoventrally broad element. Its lateral surface has a saddle-shaped profile, concave along the anterodorsal-posteroventral axis. Dorsally, the squamosal contacts the supratemporal, and anteriorly it overlies the postorbital and contributes to the posterodorsal orbital margin. The lateral wall of the dorsal quadrate articulation is formed by the squamosal (Fig. 3F–G).

#### Postorbital

The postorbital is an anterolaterally-directed, C-shaped element lying within the orbit (i.e., not forming part of the lateral temporal region). This bone is separated from the cheek region by a sharp ridge, separating the circumorbital area from the temporal region (Fig. 3C–E). The postorbital has a small dorsal exposure (Fig. 3A–B). It contacts the postfrontal, supratemporal, and quadratojugal, and is overlain by the squamosal.

#### Jugal

The jugal forms the ventral margin of the orbit. In lateral view, its anterior end is narrow and extends between the maxilla and the lacrimal to a point approximately in alignment with the anterior margin of the orbit. Posteriorly, it is overlapped by the postorbital (Fig. 3C–E).

#### Quadratojugal

Ventral to the squamosal is a small piece of bone that may represent a partial quadratojugal (Fig. 3F–G). Without exception, the quadratojugal articulates with the ventral occipital lamella of the quadrate in ichthyosaurs. In SMNS 90699, there is no element that could fulfill that role. There is, however, a small element facing posteriorly, dorsally articulating with the squamosal and laterally with the postorbital. We assume that this element is the quadratojugal, and that the descending process is broken.

#### Quadrate

The dorsal occipital lamella of the quadrate is exposed on the posterior surface of the left side of the skull (Fig. 3F). There is a higher degree of lateral curvature in the occipital lamella than is seen in the quadrate of *Ichthyosaurus* [25], but less than seen in *Eurhinosaurus* (pers. obs.). Morphology is consistent with *Stenopterygius triscissus* [26].

#### Sclerotic ring

The sclerotic ring is preserved on both sides (Fig. 3C–E), but the number of plates cannot be determined with accuracy due to poor preservation. The ring has an internal diameter of 40 mm and an external diameter of 95 mm and does not occupy the whole orbit. When the external diameter of the sclerotic ring is plotted against the diameter of the aperture standardized to orbital length, the specimen falls in the same region as Toarcian *Stenopterygius* species [27].

#### Mandible

The mandible is poorly preserved. The lateral surface is distinguished by a deep suborbital groove. The surangular and dentary are the only two elements with a significant degree of lateral exposure. The angular is present, but has no lateral exposure anterior to the initiation of the suborbital groove. In ventral view, the angular is better exposed, as is the splenial. The splenial participates in the mandibular symphysis.

#### Dentition

The teeth are small and slender, lacking macroscopic enamel ornamentation. The base of the enamel cap is poorly defined. A ring of acellular cementum separating the crown from the root appears to be present, but this was not histologically confirmed. There is no evidence for plicidentine extending as far crownward as the ring of acellular cementum (manifested as a lack of external fluting of the tooth base). The bases of the roots of most teeth remain partially enclosed by the alveolar groove.

#### Axial skeleton

The atlas-axis complex is not exposed. The first neural arch preserved in articulation with the vertebral column is assumed to be the axial neural arch, and the position in the column for both the neural arches and centra is calculated based on this assumption.

Contact between the dorsal surface of the centrum and the diapophysis is maintained until at least centrum 12 (the state in centrum 13–14 is uncertain). Based on the fusion of the di- and parapophyses, there are 44 presacral centra. An abrupt reduction in rib length and the location of the pelvic elements correlate with this anatomical marker (Fig. 2). More posterior caudal centra are also preserved, but these are not in articulation. No post-apical centra are present. Centrum length is highly variable, but generally increases from anterior to posterior, from 14–18 mm in the anteriormost dorsal region to a maximum of 27 mm in the posterior dorsal region (Fig. S2). Centrum length begins to decrease immediately anterior to the sacrum.

The cervical neural processes are tall and narrow, and the spines are posteriorly offset from the neural arches. A single neural arch is well-preserved in anterior view (centrum 14), and the anterior zygapophysis is unpaired. The spines become broader in the mid-dorsal region and less offset. In the posterior dorsal region, the spines begin to lose height, but remain essentially rectangular. At a point roughly corresponding to the sacral region, the neural spines become dorsally rounded. Contact with the preceding and following neural arch is not lost in the most posterior preserved region of the presacral column. Few postsacral neural spines are preserved, but these are strongly posteriorly inclined and rod-shaped, as described for *Stenopterygius quadriscissus* [28]. The neural processes initially decrease in height in the anterior dorsal region, before beginning to increase in height. Neural processes maintain an average height of around 71 mm until about centrum 26, at which point the process height begins to steadily decline (Fig. S2). This is also consistent with what has been described for *Stenopterygius* [28].

Corresponding to the changing morphology of the neural spines, the presacral column is subtly flexed (Fig. 2). The sacral region represents an inflection point, where the column becomes extended, and so the preflexural column is gently S-shaped.

#### Pectoral girdle

The interclavicle is T-shaped (Fig. 4, Fig. S3). The median stem is equal in length to the length of the intercoracoid suture and lacks the spatulate expansion seen in most Toarcian specimens of *Stenopterygius* [29].

The left clavicle is preserved in ventral view (Fig. 4). It lies dorsal to the acromion process of the scapula, and then extends dorsally along the anterior surface of the distal shaft of the scapula. This relationship between the acromion process and the clavicle is unexpected, since the clavicle is usually reconstructed as running ventral to the acromion process [29], and suggests that the clavicle may have enclosed the acromion but have been displaced dorsally post-mortem.

The coracoids are rectangular, with the intercoracoid facet running parallel to the long axis (Fig. 4). Three dimensionally, the coracoid is saddle-shaped in ventral view, with both the posterior glenoid region and anterior intercoracoid facet projecting further ventrally than the body of the element. Anterior coracoid notches are deep and are directed anterolaterally, parallel to the facet for articulation with the scapula. Posterior notches are present but shallow and essentially lateral in orientation. The glenoid facet of the coracoid is double the length of the scapular contribution. The scapular and glenoid facets of the coracoid are set off at an angle of approximately 110°. Although Johnson [29] described the coracoids of *Stenopterygius* as approximately equal in length and width, occasionally slightly longer than wide, the specimen described here has coracoids approximately twice as long as wide. The lack of a complex three-dimensional shape as well as the higher proportional width of the material described by Johnson [29] can probably be attributed to the strong compression undergone by most of the specimens in her study.

The distal shaft of the scapula is not prepared. The scapula has a broadly expanded proximal blade, medially differentiated into five distinct regions: a large anterior acromion process that is concave in ventral view, an anterior medial coracoid facet, a concave area edged by finished bone forming the anterolateral margin of the anterior notch, a posterior coracoid facet, and the glenoid contribution (Fig. 4). The posterior coracoid and glenoid facets of the scapula are not as offset as the corresponding facets of the coracoid; the angle between them is greater than 160°. The lateral area of the proximal blade is concave in ventral view, and the glenoid contribution is greatly thickened.

#### Forelimb

Both humeri are preserved, the right in dorsal view and the left in ventral view (Fig. 4): the left is not sufficiently prepared to provide much anatomical information. The dorsal process is narrow, forming an anterodistally trending ridge along the dorsal humerus. The proximal surface is covered with roughened bone, suggesting a cartilaginous cap, but this texture does not extend distally. A concave area occurs anterior to the dorsal process. The anterior distal humeral shaft bears a leading edge facet. Two distinct distal articular facets are present for articulation with the radius and ulna. These are approximately equal in length and appear deeply concave in dorsal or ventral view.

The zeugopodial elements are also preserved. The radius is anteroposteriorly narrower than the ulna and bears a prominent notch on its leading edge in both the right and left limbs (Fig. 4). Posteriorly, the radius articulates with the ulna, and distal to this, with the intermedium. Distally, the radius articulates with the radiale. The ulna articulates with the intermedium, ulnare, and a large pisiform. The left radiale lacks an anterior notch; the state of that on the right is ambiguous. Distally, the radiale has a large facet for articulation with distal carpal (dc) 2, and posteriorly it has smaller facets for articulation with dc3 and the intermedium. The intermedium is roughly pentagonal in shape with a large distal articular facet for dc3 and a small facet posterior to this for contact with dc4. The ulnare has two distal facets, a smaller one for articulation with dc4 and a larger posterior facet for articulation with metacarpal V. Proximal to the latter, the ulnare contacts the pisiform (Fig. 4). The pisiform is diamond-shaped, smaller than the ulnare in total area, and the distal articular facets are not well defined.

The elements distal to the proximal carpal row are disarticulated and jumbled. These elements are blocky, often at least as dorsoventrally thick as proximodistally long.

#### Hind limb

Two partial pelvic elements are preserved (Fig. 2). The more posterior is interpreted as the femur. It is proximally thickened with a flat anterior margin and a constricted mid-section. The more anterior element is flattened and rounded at the preserved end. An assignment cannot be made without more information, although the ischiopubis is a possibility.

### cf. Stenopterygius sp

#### Referred specimen

SMG uncatalogued (Fig. S1). Partial skull and skeleton from basalmost Opalinuston Formation of Mohring Brick Quarry, Heiningen, Germany.

#### Remarks and description

The axial skeleton of SMG uncatalogued is relatively undistorted, but the skull has suffered severe lateral compression. It is prepared in left lateral view, and, like SMNS 90699, is heavily impregnated with gypsum. Unlike in SMNS 90699, the postorbital is exposed on the lateral temporal region because the squamosal does not extend to the orbital margin. Additionally, SMG uncatalogued has a well-developed quadratojugal (Fig. S1B). This element is much higher than wide, and lies posterior to the postorbital. It contacts the squamosal dorsally, and is clearly visible in lateral view. Although the quadratojugal is narrow, it is similar in proportion to that of *S. triscissus* [30]. However, it is much more slender, and the entire postorbital region is much reduced compared to that described in a skull attributed to *Stenopterygius* cf. *S. quadriscissus* [31], or some specimens of *S. triscissus* [26], [30] suggesting the presence of variation in this feature. The sclerotic ring is composed of 14 plates and has an internal diameter of 43 mm and an external diameter of 95 mm.

The axial skeleton consists of 45 presacral centra, based on the point of apophyseal fusion. The longest centra occur in the mid-dorsal region (23 mm long), and centrum length begins to steadily decrease posterior to the sacrum (Fig. S2). Centrum heights are also available for the mid-dorsal region, and range between 36–46 mm (centrum height: length ratios between 1.7 and 2.2). The specimen length (back of the skull to sacral region) is approximately 96 cm. Neural processes reach their maximum height of 61 mm in the mid-dorsal region of the column and begin to decrease in height a short distance posterior to this point. In general morphology, as well as overall proportions standardized to centrum length, the neural processes are similar to those described for SMNS 90699 (Fig. S2).

Based on the reduced postorbital region, SMG uncatalogued is inconsistent with *Temnodontosaurus* and *Suevoleviathan*. A referral to *Eurhinosaurus* is considered unlikely due to the apparently unreduced mandible, the shape of the quadratojugal, and the tall dorsal neural processes. Although the temporal region has not been well-described for *Hauffiopteryx*, the robust squamosal relative to the supratemporal and quadratojugal, widely separated from the postfrontal, is more consistent with *Stenopterygius* (see [30]). For these reasons, we refer SMG uncatalogued to cf. *Stenopterygius* sp. This specimen does not contribute any information to our understanding of diversity in the Aalenian, but the presence of two *Stenopterygius* specimens in the absence of any other described reptile material suggests that this genus was not rare at this locality.

### Phylogenetic Analysis

In order to investigate the phylogenetic relationships of *Stenopterygius aaleniensis*, we performed a phylogenetic analysis focusing on species-level relationships of Lower Jurassic ichthyosaurs. The analyses was done in the software package TNT [32] with the implicit enumeration search algorithm and optimized using a parsimony criterion. Branch support was calculated by bootstrapping the matrix 1000 times; values are only given for clades recovered in more than 50% of replicates. Bremer support values are also presented when greater than 1.

**Figure 5:**
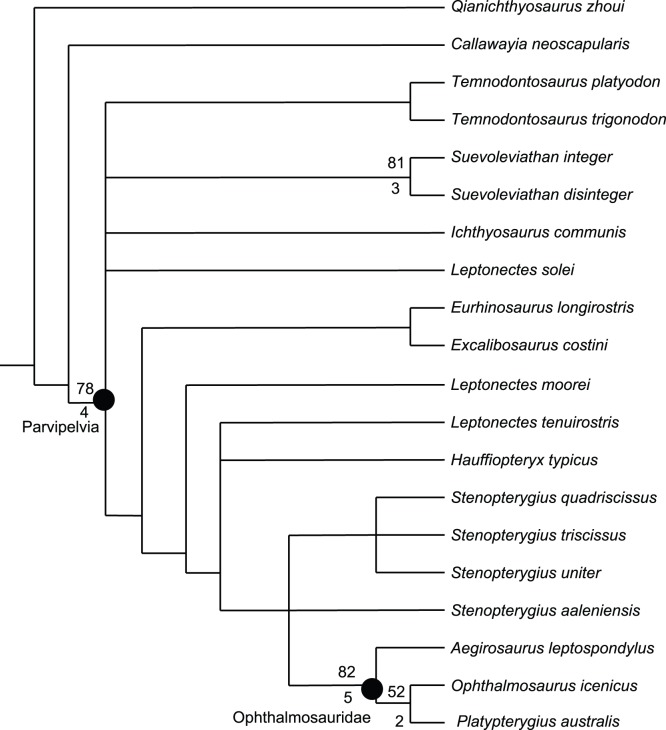
Phylogenetic relationships of Lower Jurassic ichthyosaurs. Cladogram of the strict consensus of 6 most parsimonious trees (data presented in Table S3). Length  = 138; CI = 0.493; RI = 0.675. Bootstrap support values of greater than 50% are presented above the branches, Bremer support values of greater than 1 are presented below.

The species-level analysis is considered to be superior to coding the new taxon in existing ichthyosaur matrices because it does not assume generic monophyly. The matrix consists of 60 morphological characters and 20 taxa coded from the literature and from personal observation. Character descriptions are presented in Table S2; the character by taxon matrix (generated in Mesquite [33]) is presented in Table S3, and a list of synapomorphies supporting each node is presented in Table S4. Q*ianichthyosaurus zhoui* was used as the outgroup. Six most parsimonious trees were recovered of length 138 (CI = 0.493; RI = 0.613) (strict consensus: Fig. 5). *Stenopterygius* formed the sister taxon to the Ophthalmosauridae, as in several previous ophthalmosaurid-specific analyses [34], [35] and a global analysis [11], differing from the topology recovered in [36] and subsequent reanalyses thereof. Leptonectidae (family) and *Leptonectes* (genus) were paraphyletic, forming a series of successive sister groups to *Stenopterygius* + Ophthalmosauridae. Branch support values were universally low with only the Parvipelvia (sensu [36]), *Suevoleviathan* (genus), Ophthalmosauridae and an internal ophthalmosaurid node receiving bootstrap supports of higher than 50%. The same set of nodes also had Bremer support values of >1. The analysis failed to resolve the position of *Stenopterygius aaleniensis*, placing this taxon in a polytomy with the Posidonia Shale *Stenopterygius* spp. and Ophthalmosauridae. The principal feature separating the Toarcian *Stenopterygius* spp. from *S. aaleniensis* and ambiguously supporting the association of the latter with Ophthalmosauridae is the absence of a notch on the anterior radiale (Table S4; the second feature, the well developed occipital flange of the parietal, is obscured by matrix in *S. aaleniensis*). Notching of the anterior surface of the digital elements is highly variable in ichthyosaurs within species [29], genera [2], and also phylogenetically (this study). For this reason, we follow a conservative approach and refer SMNS 90699 to *Stenopterygius* in spite of its ambiguous position in the phylogenetic analysis.

## Discussion

### Comparative Cranial Morphology – Generic Level

**Figure 6:**
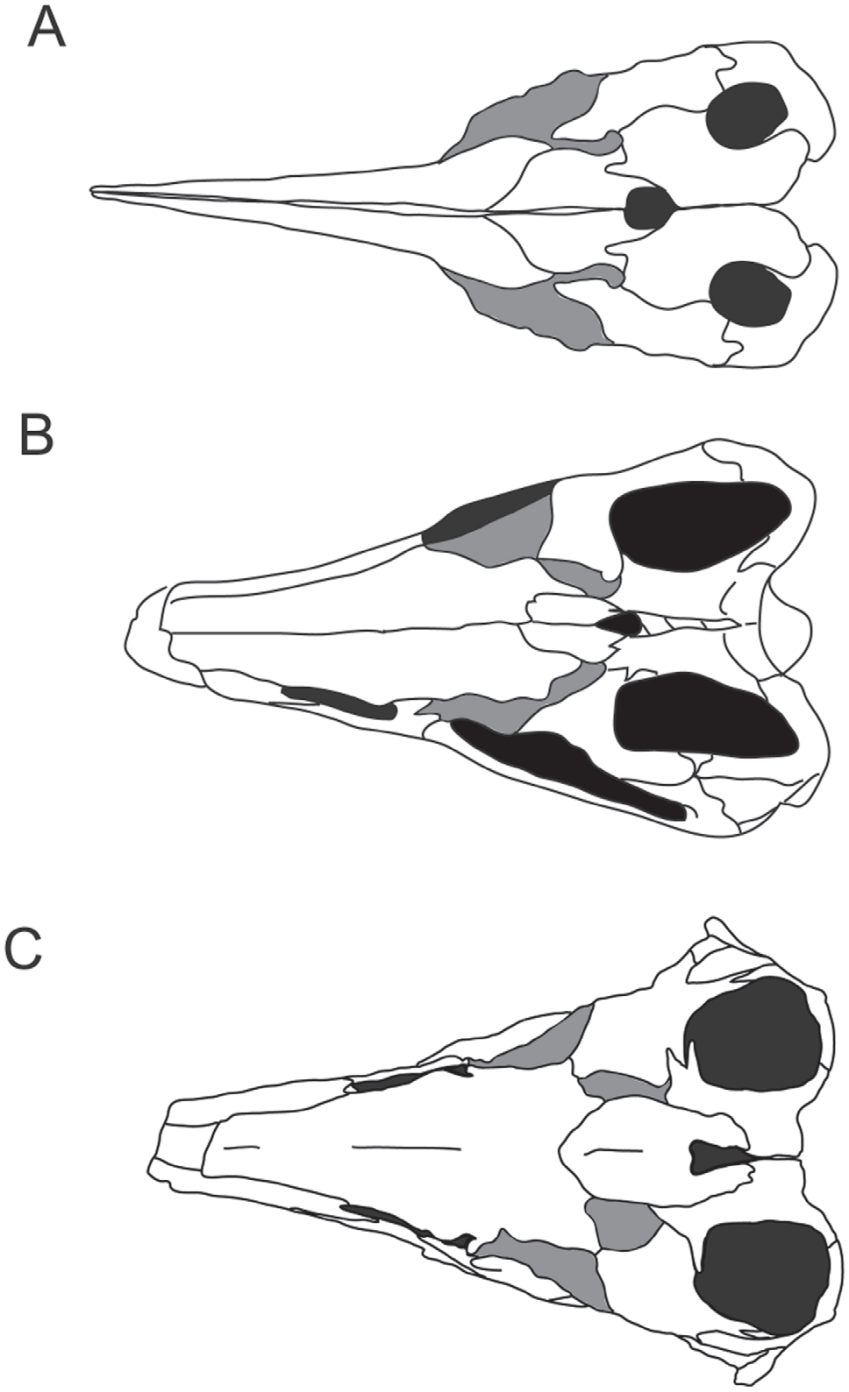
Comparative morphology of the skull roof in three ichthyosaurs. A, *Hauffiopteryx typicus* (modified from [30]); B, *Stenopterygius triscissus* (modified from [23]); C, *Stenopterygius aaleniensis* sp. nov. The prefrontal has been shaded grey to facilitate comparisons.

The cranial morphology of *Stenopterygius* is distinctive and will be discussed further. Although the exclusion of the postfrontal-frontal contact by a medial exposure of the prefrontal appears to be widely distributed in Lower Jurassic ichthyosaurs (e.g., *Ichthyosaurus* [23], *Leptonectes* [37], *Hauffiopteryx* [30]), the occurrence of a constriction separating the lateral and medial prefrontal exposures in dorsal view has a slightly more limited distribution (*Hauffiopteryx, Ichthyosaurus* and *Stenopterygius*) (Fig. 6). Caine and Benton [30] considered the presence of a contact between the nasal and parietal to be diagnostic of *Stenopterygius triscissus*, but this is very unlikely. Although present in the British material [30], this feature is absent in the skull described by Motani [23], asymmetric in that described by Mazin [26], and also variable in the material described by Godefroit [24]. Godefroit reconstructed the contact between the nasals and parietals as being extremely robust, but his specimen drawings are more consistent with the contact being only a thin spicule of bone, if present, which is consistent with other authors [24], [38]. This type of asymmetry and variability in the sutural contact between the nasal and parietal of a single species is also seen in *Leptonectes tenuirostris* [2], [37], [39]. Likewise, the presence and degree of contact between the nasals and postfrontals appears to be variable.

In dorsal view, the parietal does not play a significant role in the anterior edge of the upper temporal fenestra in *Stenopterygius*, unlike in *Leptonectes* and *Hauffiopteryx* [30], [37]. Possibly correlated to this, the postfrontal has a broad, fan-shaped exposure anterior to the upper temporal fenestra, unlike in *Hauffiopteryx* where its contribution to the edge of the temporal fenestra is minimal [30]. The frontals are strongly convex immediately anterior to the parietal foramen [26], [30]. The prefrontals have two regions of exposure on the dorsal skull roof, in a supraorbital position and lateral to the frontals [23].

The temporal region is also of interest for generic referral. The postorbital region is relatively reduced in *Stenopterygius* and has a strong posterior curvature [26]. This is very different from *Temnodontosaurus, Ichthyosaurus* and *Suevoleviathan,* where the elements of the cheek (dorsal quadratojugal, squamosal when present) are best observed in lateral view. A prominent squamosal is typically present in *Stenopterygius* [30]. The lateral ramus of the supratemporal is slender [26], contrary to the state in *Leptonectes* and *Hauffiopteryx* where it is the most prominent element of the posterior skull [30], [37].

### Specific Referral

Maxwell [40] devised a metric scheme for distinguishing the species of *Stenopterygius* from the early Toarcian of southwestern Germany, and recognized three valid species: *S. quadriscissus, S. triscissus,* and *S. uniter*. However, the suggested criteria are extremely difficult to apply to a three-dimensionally preserved specimen in which both the anterior rostrum and the hind limb are missing, as it is based on complete albeit two-dimensional material. Several authors have described three-dimensional cranial material referable to *Stenopterygius* from the UK, Belgium, and France [24], [26], [30] – in all cases, the specimens have been assigned to *S. triscissus* (formerly *Stenopterygius longifrons* [41]). No three-dimensionally preserved cranial material has been reported for *S. quadriscissus* or for *S. uniter*.

The skull of SMNS 90699 differs from the skulls referred to other species of *Stenopterygius* in that the internasal depression is extremely reduced, and a groove between the anterior frontals is absent. The upper temporal fenestrae are antero-posteriorly shorter and more rounded than in other species (Fig. 6). In most of the Toarcian *Stenopterygius* material, the long axis of the external narial opening is approximately parallel to the long axis of the rostrum, whereas in the Aalenian specimen, the posterior edge is deflected dorsally, more similar to the bilobate external narial opening described for *Ophthalmosaurus* [5]. The maxilla extends further under the orbit than in other *Stenopterygius* species – tooth positions situated under the orbit are never observed in adult early Toarcian *Stenopterygius* material, whereas in the Aalenian taxon they are clearly present. Also, the cheek region is more posteriorly directed than in the cranial material referred to *S. triscissus*, and the lateral projection of the postorbital and squamosal to support the eye is more prominent and less anteriorly directed [26].

The postcranial skeleton is generally consistent with *Stenopterygius*, but some differences are observed. The radius and ulna share a short articular facet, which is uncommon in Toarcian specimens. In addition, the absence of notching of the anterior radiale, even as an asymmetry, is a feature that is completely unknown in the Toarcian species and *S. cayi* [7]. The phalanges are greatly thickened, a feature shared with *S. cayi* and probably also with *S. quadriscissus*. Maisch and Matzke [11] proposed this feature as an ophthalmosaurid synapomorphy, but Fischer et al. [34] optimized it as a synapomorphy of the clade *S. cayi* + Ophthalmosauridae [9].

Well-ossified epiphyses of the humerus, radius, and ulna [42], fusion of cranial sutures on the dorsal skull, and partial fusion of some of the neural arches and centra suggest that SMNS 90699 was an osteologically mature adult. Based on the length of the humerus (101 mm), this specimen is in the size range of large adults of *Stenopterygius quadriscissus* and *S. triscissus*, or small individuals of *S. uniter*.

These differences, both in the cranial and postcranial skeleton, combined with a stratigraphic gap of approximately 6 million years between the youngest Posidonia Shale specimen and SMNS 90699 [43], lead us to propose a new species. However, based on a poor understanding of the effects of taphonomic deformation on perceived skull morphology in *Stenopterygius*, in the future it may become obvious that *S. aaleniensis* is referable to one of the existing species, or is present but unrecognized in the Posidonia Shale.

### Stenopterygius Cayi

The Bajocian-aged *Stenopterygius cayi* is the youngest species referable to the genus *Stenopterygius. Stenopterygius cayi* shares dorsoventrally thickened phalanges with *S. aaleniensis*, as well as general characteristics common to all *Stenopterygius* species (polygonal metacarpals, forefin bearing four ossified digits, anterior margin of radius notched: all symplesiomorphic for *Stenopterygius* in the new analysis) [7]. *S. cayi* differs from *S. aaleniensis* in several respects, most notably in size, but also in the shape of the interclavicle, which is broadly expanded at the intersection of the transverse and median bars (Fig. S3). The radiale in *S. cayi* also bears an anterior notch [7], optimized as a synapomorphy uniting the Toarcian species in the new analysis. A notched radiale is absent in *S. aaleniensis*.

The position of *Stenopterygius cayi* in the analyses of Fischer and colleagues [9], [34] requires further discussion, since it suggests that the genus *Stenopterygius* as defined here is paraphyletic. This is relevant to the current contribution because the thickened phalanges seen in *S. aaleniensis* are one of two synapomorphies suggesting a close relationship between *S. cayi* and Ophthalmosauridae. Although the paddle elements of both *S. cayi* and *S. aaleniensis* are thickened, as in Ophthalmosauridae, they are most consistent in shape with other species of the genus *Stenopterygius,* being closely packed and anteroposteriorly elongate proximally and becoming loosely packed and more rounded distally. The second character cited by Fischer et al., absence of a ventral notch on the basioccipital, was coded as present for *Stenopterygius quadriscissus*, but appears to be absent in the majority of specimens ([14], [44], pers. obs.). We consider both *S. cayi* and *S. aaleniensis* to be referable to *Stenopterygius* until stronger evidence linking them to ophthalmosaurids is forthcoming.

### Size

Ichthyosaur material from the late Toarcian – Bajocian referred to *Stenopterygius* is typically of much larger body size than *S. quadriscissus*, regardless of the metric considered. For example, a skull reported from the late Toarcian of France has a premaxilla that is 45% longer than the largest specimen referred to *S. quadriscissus*, and also longer than the longest premaxilla referred to *S. triscissus* (by 9%) and *S. uniter* (by 12%) [45]. Likewise, with a jaw length of 990 mm, *Stenopterygius cayi* dwarfs the largest *S. quadriscissus* specimen (MHH 1981/33– jaw length: 564 mm; *S. triscissus*: 675 mm –MHH 1b), and even the larger *S. uniter* (PMU R154– jaw length: 721 mm) [7]. In fact, most ichthyosaur specimens reported from this time interval tend to exceed early Toarcian *Stenopterygius* species in size (e.g., *Mollesaurus* [8], vertebrae reported from the Aalenian of France [15], a *Temnodontosaurus-*like ichthyosaur from the late Toarcian of France [46]). This is interesting because the smaller genera (*Hauffiopteryx*, *Stenopterygius*) are by far the most abundant specimens found in the Posidonia Shale. It has previously been hypothesized that *Stenopterygius* underwent a body size increase between the early and late Toarcian [45], but a literal interpretation of published range data [2], [41] implies a faunal turnover, with the numerically dominant small- to mid-sized ichthyosaur species reduced in taxonomic diversity or eliminated. The finds from Heiningen are important in that they are not only diagnostic, but represent the last Jurassic occurrence of an ichthyosaur with an adult form under 4 m in length. It has been suggested that some Cretaceous taxa re-colonized this body size range in the Early Albian, approximately 65 million years later [35], [47].

### Aalenian-Bathonian Interval

Prior to the current contribution, articulated ichthyosaur material from the Aalenian–Bathonian interval was known only from western Argentina [1], [7], [8]. This gap led to the suggestion that the Toarcian-Aalenian boundary represented an extinction event among ichthyosaurs, with two groups, *Stenopterygius*-like taxa and *Leptonectes*-like taxa disappearing suddenly around this time [48]. This interval also represents a time period during which the guild structure of marine ecosystems changed, with many niches formerly occupied by ichthyosaurs being filled with pliosauroids and crocodiles [49]. The current contribution represents the first recorded occurrence of *Stenopterygius* in the Aalenian, and reduces the length of the ghost range between the early-middle Toarcian *Stenopterygius* species from Europe, and *Stenopterygius cayi* from the Bajocian of Argentina.

In spite of the perceived loss of ecological dominance by ichthyosaurs after the Toarcian [49], none of the three main groups of marine reptiles have a strong Aalenian record. Marine crocodiles, plesiosaurs, and ichthyosaurs all show a diversity minimum in the Aalenian, and evidence suggests an underestimation of taxonomic diversity during this interval [3], [4]. Only fragmentary plesiosaurian remains have been described from the Aalenian of France and Germany (summarized by [50]), and teleosaur remains attributed to *Steneosaurus* are known from South Dagestan [51]. It is likely that the transition in ecological structure between the major groups of marine reptiles [49], as well as the diversification of the ophthalmosaurids and extinction of the basal ichthyosaur lineages, was more gradual than often portrayed in the literature (e.g., [48]). The shortage of productive Aalenian – Bathonian localities creates a skewed picture of ecological and evolutionary processes during this interval.

### Conclusions

SMG uncatalogued and SMNS 90699 represent the most complete and well-preserved marine reptiles from the Aalenian, and are the only ichthyosaurs from this time interval that are diagnostic to genus or species level. Although the more complete of the two, SMNS 90699, demonstrates some unique character combinations, it is referable to the Toarcian genus *Stenopterygius* based on similarities in cranial morphology and forelimb structure. These specimens provide data for a critical time period in ichthyosaur evolution, between the taxonomically and ecologically diverse ichthyosaur faunas of the Toarcian (five unrelated genera, filling different ecological roles based on dental morphology and morphology of the axial skeleton: [28], [49]), and the ecologically and taxonomically impoverished ichthyosaur fauna of the Callovian (a single species [2]). More data, especially from the marine crocodiles and plesiosaurs that replaced ichthyosaurs in abundance and diversity, is needed to gain a better understanding of this transition.

## Supporting Information

Figure S1 **SMG uncatalogued.** A, photograph of the presacral portion of the specimen. B, Interpretation of skull elements.
(TIF)

Figure S2 **Graphs of vertebral length and neural process height for SMG uncatalogued and SMNS 90699**, based on the data in Table S1.
(EPS)

Figure S3 **Interclavicle.** Of A, SMNS 90699, *Stenopterygius aaleniensis* holotype. B, MOZ 5803, *S. cayi* holotype (posterior portion of medial bar broken).
(TIF)

Table S1 **Raw data used to construct Figure S2.** Measurements in millimeters.
(PDF)

Table S2 **Characters used in the phylogenetic analysis.**
(DOC)

Table S3 **Character by taxon matrix used in the phylogenetic analysis.**
(NEX)

Table S4 **Optimization of synapomorphies, based on the clades recovered in** **Fig. 5** **.**
(DOC)
