# Supplementary figures and images for: First Diagnostic Marine Reptile Remains from the Aalenian (Middle Jurassic): A New Ichthyosaur from Southwestern Germany
Source: PLoS One. 2012 Aug 1;7(8):e41692. doi: 10.1371/journal.pone.0041692 (PMC3411580; doi:10.1371/journal.pone.0041692)

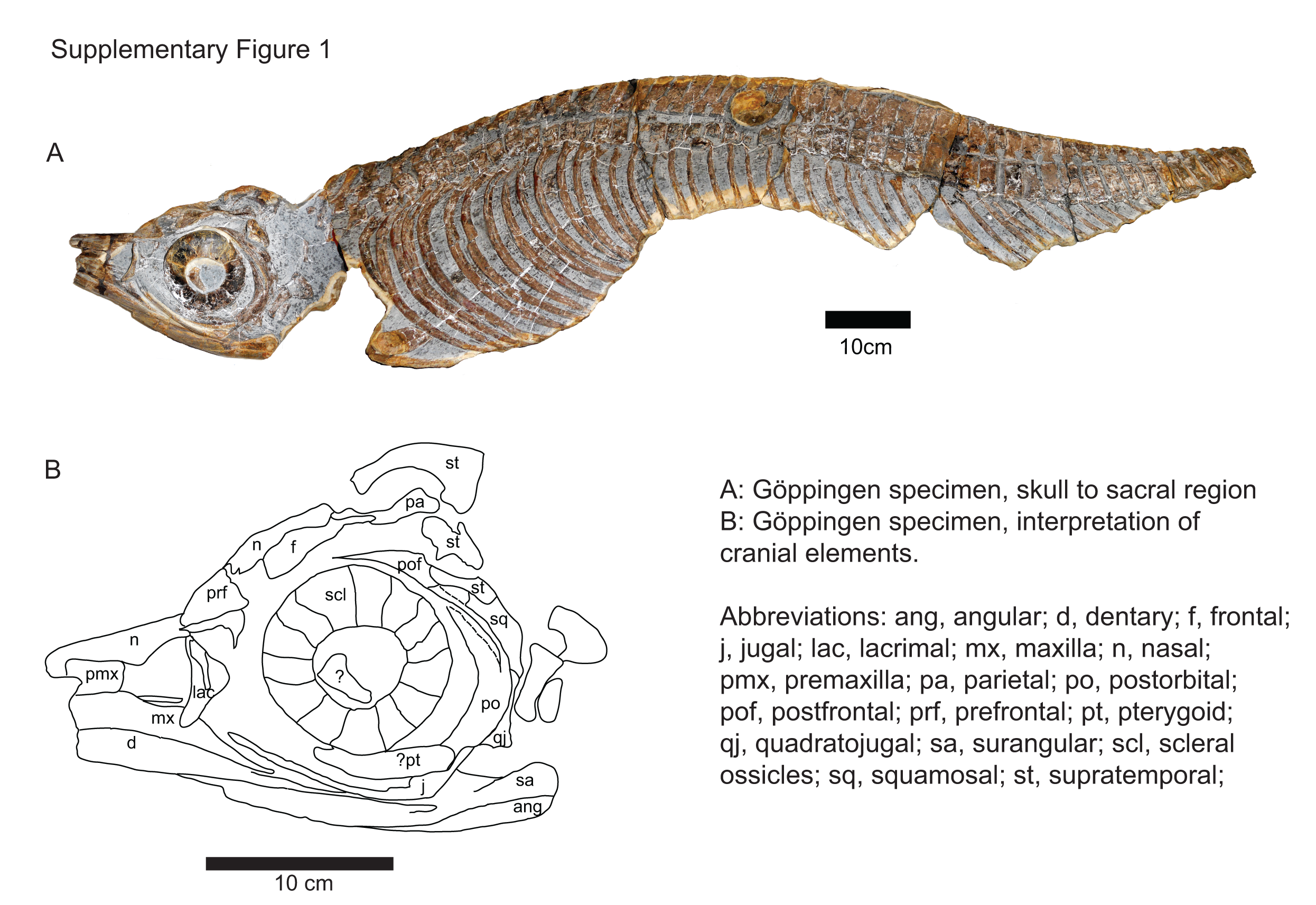

Supplement: Figure S1 — SMG uncatalogued. A, photograph of the presacral portion of the specimen. B, Interpretation of skull elements. (TIF) [file pone.0041692.s001.tif]

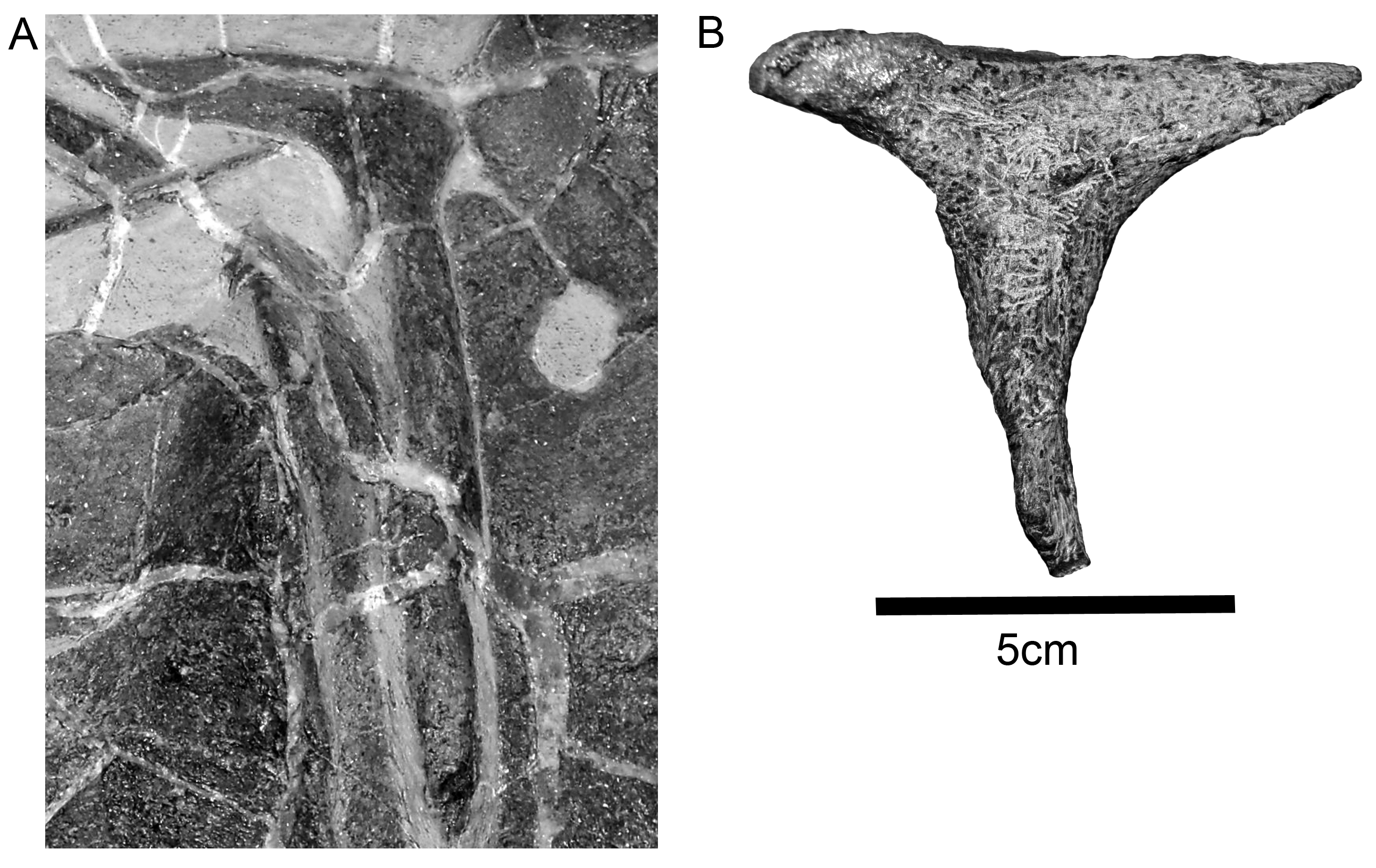

Supplement: Figure S3 — Interclavicle. Of A, SMNS 90699, Stenopterygius aaleniensis holotype. B, MOZ 5803, S. cayi holotype (posterior portion of medial bar broken). (TIF) [file pone.0041692.s003.tif]
