# Supplementary material for: First Diagnostic Marine Reptile Remains from the Aalenian (Middle Jurassic): A New Ichthyosaur from Southwestern Germany
Source: PLoS One. 2012 Aug 1;7(8):e41692. doi: 10.1371/journal.pone.0041692 (PMC3411580; doi:10.1371/journal.pone.0041692)
Supplement: Table S1 — Raw data used to construct Figure S2. Measurements in millimeters. (PDF) [file pone.0041692.s004.pdf]

SMNS 90699

Centrum	Length	Neural process
1		
2		60
3	14	63
4	18	63
5		63
6	19	60
7	19	59
8	18	59
9		
10	15	57
11	18	55
12	20	
13	19	65
14	21	67
15	20	65
16	22	68
17		74
18	20	66
19	23	70
20	23	68
21	25	69
22	22	71
23	21	68
24	23	69
25	26	69
26	25	68
27	23	65
28	25	65
29	22	63
30	24	65
31	23	61
32	27	61
33	24	58
34	26	
35	26	56
36	24	55
37	26	55
38	25	54
39	26	51
40	24	48
41	27	46
42	26	47
43	25	43
44	25	43
45	24	43
46	23	42

SMG uncatalogued

Centrum	Length	Height	Neural process
1			
2			
3	15		
4	16		
5	16		
6	16		
7	18		41
8	15		
9	19		
10	16		
11	18		43
12	18		46
13	19		44
14	18		47
15	19		50
16	20		49
17	19		
18			56
19	19		55
20	20		58
21	22		61
22	23		60
23	21		
24			
25	20		
26	23		
27	21	36	58
28	22	39	51
29	23	42	54
30	22	46	55
31	21	47	56
32	22	47	54
33	21	46	53
34	20		51
35			
36	21		49
37	19		45
38	20		42
39	21		39
40	21		38
41			38
42	21		36
43	21		31
44	21		
45	20		
46			

47	23	36
48	25	
49		
50		
51		
52		
53		
54		
55		
56		
57		
58		
59		
60		
61		
62		
63		
64		
65		
66		
67		
68		
69		
70		
71		
72		

47	18
48	19
49	
50	19
51	19
52	
53	18
54	19
55	17
56	17
57	17
58	17
59	17
60	18
61	17
62	
63	17
64	16
65	15
66	15
67	15
68	14
69	14
70	13
71	
72	11
