# Supplementary material for: First Diagnostic Marine Reptile Remains from the Aalenian (Middle Jurassic): A New Ichthyosaur from Southwestern Germany
Source: PLoS One. 2012 Aug 1;7(8):e41692. doi: 10.1371/journal.pone.0041692 (PMC3411580; doi:10.1371/journal.pone.0041692)
Supplement: Table S2 — Characters used in the phylogenetic analysis. (DOC) [file pone.0041692.s005.doc]

Supplementary Information Table 2:

Phylogenetic analysis – character list

**Skull**

1. Premaxilla: (0) supra- and sub-narial processes absent; (1) well-developed supranarial process overlying approximately 1/3–1/2 of external nares; (2) supranarial process short relative to subnarial process, or both processes short relative to external narial length.
2. Maxilla, lateral view: (0) premaxillary process short, extending approximately 1 narial lengths or less anterior to external narial opening; (1) premaxillary process long, extending approximately 1.5 narial lengths or more anterior to external nares. [modified from Fernandez 2007: character 1]
3. Maxilla, jugal process: (0) long, extending as far under orbit as lacrimal in lateral view; (1) short, hidden from lateral view by jugal.
4. Nasals, dorsal exposure: (0) substantial, dorsally exposed over more than 40% or more of the prenarial rostrum; (1) reduced, dorsally exposed over less than 40% prenarial rostral length.
5. Lacrimal. (0) excluded from contact with external nares by dorsal process of maxilla, (1) participates in external narial opening [Sander 2000: character 10].
6. Postorbital: (0) contacting supratemporal in external view; (1) excluded from contact with supratemporal [modified from Sander 2000: character 27].
7. Jugal, dorsal ramus: (0) well-developed and strongly curved dorsally; (1) poorly developed, jugal essentially straight.
8. Quadratojugal: (0) broadly exposed in lateral view, (1) quadratojugal narrow and most extensively exposed in posterior view. [modified from Motani 1999: character 25]
9. Quadratojugal: (0) ventral edge concave, resulting in an embayment anterior to the quadrate articulation; (1) ventral edge straight or entirely covered by jugal.
10. Squamosal, descending process: (0) short, not extending the entire length of the posterior quadratojugal; (1) long, extending as far ventrally as the quadrate – quadratojugal articulation.
11. Squamosal, dorsal quadrate articulation: (0) dorsal quadrate enclosed laterally by squamosal; (1) lateral wall absent, quadrate articulates dorsomedially with supratemporal only.
12. Prefrontal, dorsal view: (0) absence of exposure on dorsal skull roof anterior to parietal, (1) exposure present but limited by anterior process of postfrontal and posterior process of nasal, (2) broadly exposed.
13. Frontals, dorsal view: (0) strongly convex at anterior edge of parietal foramen, (1) flat to concave, overlapped by surrounding elements.
14. Parietal, dorsal view: (0) with no contribution to anterior edge of UTF, postfrontal excluded by frontal; (1) with minor contribution to anterior edge of UTF, (2) large contribution, almost completely excluding postfrontal from contact with anterior edge of UTF.
15. Parietal foramen: (0) situated at a level approximately equal to anterior edge of UTF, (1) situated well anterior to anterior edge of UTF.
16. Parietal, occipital flange: (0) medial flange present and large in posterior view; (1) reduced or absent in posterior view.
17. Supratemporal, descending ramus: (0) pronounced, reaching at least half of the total height of the quadrate; (1) reduced, a small process medial to the quadrate articulation [modified from Motani 1999: character 22].
18. Stapes, posterior view. Lateral head notably less robust than medial head (0), both medial and lateral heads robust (1) [modified from Sander 2000: character 34].
19. Supraoccipital: (0) exoccipital processes parallel, (1) exoccipital processes divergent.
20. Exoccipitals: (0) exoccipitals make up the largest lateral contribution to the foramen magnum, (1) supraoccipital contribution almost equal to that of exoccipitals.
21. Basioccipital, contribution to foramen magnum: (0) minimal/absent, almost entirely excluded by exoccipitals and a sagittal crista on its dorsal surface; (1) present, the portion contributing to the floor of foramen magnum flat and covered in finished bone.
22. Basioccipital, anterior peg: (0) large, approximately as long as the anteroposterior length of the condyle in dorsal view; (1) reduced [modified from Motani 1999: character 29]
23. Basioccipital: (0) ventral extracondylar area larger than dorsoventral height of condyle, (1) ventral extracondylar area smaller than condyle but still extensive, (2) ventral extracondylar area reduced to a thin strip.
24. Basioccipital: (0) basioccipital forming prominent ventral tubera separated by a deep notch, (1) tubera reduced or absent [modified from Fischer et al. 2012: character 19]
25. Palatine: (0) posterior palatine forming the anterior edge of the subtemporal fenestra, (1) posterior palatine excluded from the subtemporal fenestra by the postpalatine process of the pterygoid [modified from Maisch and Matzke 2000: character 38]
26. Lower jaw: (0) well-developed, (1) reduced relative to upper jaw [modified from Motani 1999: character 33]
27. Splenial: (0) splenial playing a more extensive role in the mandibular symphysis than the dentary, (1) splenial participating in the mandibular symphysis but restricted to the posterior half [modified from Sander 2000: character 39]
28. Angular, anterior lateral exposure: (0) minimal, terminating posterior to the anteriormost exposure of surangular; (1) well-exposed, extending as far anterior as surangular [modified from Motani 1999: character 32]
29. Angular, posterior lateral exposure: (0) minimal, less than half the lateral surface of the retroarticular process is covered by the angular, (1) extensive, surangular exposure reduced to a thin strip on the lateral surface of the retroarticular process.
30. Dentition: (0) enamel with prominent ridges and grooves, (1) enamel thin and essentially smooth, (2) enamel thick and bumpy [modified from Druckenmiller and Maxwell 2010: character 25]

**Forelimb**

Note: all notching characters were coded as invariant if one state is present in over 80% of all specimens examined.

1. Scapula: (0) with prominent acromion process, (1) large acromion process absent but anterior expansion of proximal scapula present, (2) proximal expansion absent, scapula strap-shaped.
2. Coracoid: (0) mediolaterally wider than anteroposteriorly long, (1) approximately equidimensional, (2) much anteroposteriorly longer than mediolaterally wide.
3. Coracoid: (0) with well-developed anterior notch, (1) anterior notch reduced or absent, coracoid essentially round to ovate.
4. Coracoid: (0) with well-developed posterior notch, such that the glenoid facet is offset from the medial portion of the element; (1) poorly developed or absent posterior notch.
5. Humerus: (0) without distal facet for preaxial accessory; (1) anterior distal facet for accessory element present. [Godefroit 1993: character 10]
6. Humerus: (0) facets for radius and ulna essentially continuous in adult, (1) distal humeral facets separated by a notch.
7. Preaxial digit: (0) no additional element anterodistal to the radius giving rise to a preaxial digit, (1) preaxial digit present as described above [Motani 1999: character 75]
8. Radius: (0) with anterior notch, (1) anterior notch absent [modified from Motani 1999: character 59]
9. Radius: (0) posterior notch present, (1) absent. [Motani 1999: character 60]
10. Ulna: (0) anterior notch present, (1) absent. [Motani 1999: character 63]
11. Radiale: (0) anterior notch present; (1) absent. [Motani 1999: character 65]
12. Manual digit II: (0) notching on elements of anterior digit distal to metacarpal II, (1) notching of distal elements absent [Motani 1999: character 71]
13. Intermedium: (0) two subequal distal facets supporting digits III and IV, (1) with one distal facet much larger than the other supporting digit III [modified from Sander 2000: character 93]
14. Ulnare: (0) with one distal facet, primarily supporting a single digit; (1) with a posteriorly oriented distal facet for articulation with metacarpal V. [Note: taxa in which the ulnare has a distal articulation for metacarpal V but lacks a posterodistal facet, e.g. *Ichthyosaurus* and *Ophthalmosaurus*, are coded as 0]
15. Manual digits: (0) four or fewer ossified elements in the metacarpal row, (1) five or more ossified elements in the metacarpal row [modified from Sander 2000: character 95]
16. Metacarpals: (0) rounded, not forming tightly interlocking mosaic, (1) polygonal, forming a tightly interlocking mosaic.

**Hindlimb**

Note: all notching characters were coded as invariant if one state is present in over 80% of all specimens examined.

1. Ilium: (0) with expanded proximal region, (1) narrow proximally and distally, rib-like (1) [modified from Sander 2000: character 106]
2. Pubis: (0) with obturator foramen mostly in pubis; (1) part of obturator fossa [modified from Motani 1999: character 84]
3. Ischium and pubis relative size: (0) approximately equal in size, (1) ischium much larger than pubis [modified from Sander 2000: character 105]
4. Ischium and pubis: (0) medially fused, (1) medially separate [modified from Motani 1999: character 83]
5. Ischium and pubis: (0) laterally fused, (1) laterally separate [modified from Motani 1999: character 83]
6. Ischium: (0) with significantly greater acetabular contribution than pubis, (1) subequal contribution.
7. Ischium: (0) with greatly expanded medial edge, (1) ischium lacking medial expansion.
8. Hind limb reduction: (0) distal limb more than double the length of the femur, (1) distal limb shortened.
9. Tibia: (0) approximately twice as proximodistally long than more distal elements, (1) tibia only slightly longer than successive elements [modified from Motani 1999: character 90]
10. Tibia and fibula – relative size: (0) tibia larger than fibula; (1) approximately equal in size; (2) tibia anterioposteriorly and proximodistally smaller than fibula.
11. Proximal tibia and fibula: (0) separated by a gap, (1) in contact [modified from Motani 1999: character 88].
12. Tibia: (0) anterior notch present; (1) absent [Motani 1999: character 92]
13. Fibula: (0) anterior notch present, (1) absent.
14. Pes, digit II: (0) elements distal to the tibia notched, (1) notching absent.

**References**

Druckenmiller, P. S., and E. E. Maxwell. 2010. A new Lower Cretaceous (lower Albian) ichthyosaur from the Clearwater Formation, Alberta, Canada. Canadian Journal of Earth Sciences 47:1037-1053.

Fernández, M. S. 2007. Redescription and phylogenetic position of *Caypullisaurus* (Ichthyosauria: Ophthalmosauridae). Journal of Paleontology 81:368-375.

Fischer, V., M. W. Maisch, D. Naish, R. Kosma, J. Liston, U. Joger, F. J. Krüger, J. Pardo Pérez, J. Tainsh, and R. M. Appleby. 2012. New ophthalmosaurid ichthyosaurs from the European Lower Cretaceous demonstrate extensive ichthyosaur survival across the Jurassic-Cretaceous boundary. PLoS One 7:e29234.

Godefroit, P. 1993. The skull of *Stenopterygius longifrons* (Owen, 1881). Revue de Paléobiologie 7:67-84.

Maisch, M. W., and A. T. Matzke. 2000. The Ichthyosauria. Stuttgarter Beiträge zur Naturkunde Serie B (Geologie und Paläontologie) 298:1-159.

Motani, R. 1999. Phylogeny of the Ichthyopterygia. Journal of Vertebrate Paleontology 19:472-495.

Sander, P. M. 2000. Ichthyosauria: their diversity, distribution and phylogeny. Paläontologische Zeitschrift 74:1-35.
