# Supplementary material for: First Diagnostic Marine Reptile Remains from the Aalenian (Middle Jurassic): A New Ichthyosaur from Southwestern Germany
Source: PLoS One. 2012 Aug 1;7(8):e41692. doi: 10.1371/journal.pone.0041692 (PMC3411580; doi:10.1371/journal.pone.0041692)
Supplement: Table S4 — Optimization of synapomorphies, based on the clades recovered in Fig. 5 . (DOC) [file pone.0041692.s007.doc]

SI Table 3: List of synapomorphies recovered based on the analysis of the matrix presented in SI Table 2. Note the high degree of homoplasy.

| Node | Synapomorphies |
| --- | --- |
| Parvipelvia | 48: obturator foramen not mostly enclosed by pubis  55: tibia only slightly longer than more distal elements  59: fibula lacking an anterior notch (contiguous shaft of Motani 1999) |
| *Suevoleviathan* | 10: elongated descending process of squamosal  30: enamel thick, with bumpy ornamentation  44: ulnare with posteriorly oriented distal facet for articulation with mcV  47: proximally expanded ilium |
| *Temnodontosaurus* | 2: elongate maxilla in lateral view  7: poorly developed dorsal ramus of jugal  46: metacarpals polygonal |
| Clade including both *Eurhinosaurus* + *Ophthalmosaurus* | None |
| *Eurhinosaurus* + *Excalibosaurus* | 26: reduced lower jaw |
| Clade including *Leptonectes moorei* + *Ophthalmosaurus* | 44: ulnare with posteriorly oriented distal facet for articulation with mcV  46: metacarpals polygonal |
| Clade including *Leptonectes tenuirostris* + *Ophthalmosaurus* | 31: scapula with well-developed acromion process |
| Clade including *Stenopterygius* + *Ophthalmosaurus* | 1: premaxilla with reduced supranarial process  51: ischium and pubis laterally fused |
| Toarcian *Stenopterygius* spp. | 16: medial occipital flange of parietal well-developed  41: radiale notched |
| Ophthalmosauridae | 3: jugal process of maxilla short in lateral view  12: Lack of prefrontal exposure on the dorsal skull roof anterior to the parietal  30: enamel ornamented with pronounced ridges  37: preaxial digit present anterior to digit II  38: loss of anterior (peripheral) notch on radius  45: more than 5 ossified metacarpals  60: loss of anterior notches on the elements of digit II (pes) |
| *Ophthalmosaurus* + *Platypterygius* | 13: frontals not strongly convex  29: surangular role in retroarticular process reduced  35: preaxial facet present on distal humerus |
